# A Predictive Model for Severe COVID-19 in the Medicare Population: A Tool for Prioritizing Primary and Booster COVID-19 Vaccination

**DOI:** 10.3390/biology10111185

**Published:** 2021-11-15

**Authors:** Bettina Experton, Hassan A. Tetteh, Nicole Lurie, Peter Walker, Adrien Elena, Christopher S. Hein, Blake Schwendiman, Justin L. Vincent, Christopher R. Burrow

**Affiliations:** 1 Humetrix Inc., Del Mar, CA 90214, USA; aelena@humetrix.com (A.E.); chein@humetrix.com (C.S.H.); bschwendiman@humetrix.com (B.S.); cburrow@humetrix.com (C.R.B.); 2 Warfighter Health Mission Team, Department of Defense Joint Artificial Intelligence Center (JAIC), Arlington, VA 22202, USA; hassan_tetteh@hks09.harvard.edu; 3 Coalition for Epidemic Preparedness Innovation (CEPI), Washington, DC 20006, USA; drnickilurie@gmail.com; 4 Department of Medicine, Harvard Medical School, Boston, MA 02115, USA; 5 US Navy, Washington, DC 20376, USA; peter.b.walker.mil@mail.mil; 6 Amazon Web Services Inc., Seattle, WA 98109, USA; vinjust@amazon.com

**Keywords:** COVID-19 vaccine prioritization, COVID-19 booster vaccine, severe COVID-19 disease, risk for severe COVID-19 infection, COVID-19 vaccine booster prioritization, Medicare population, severe COVID-19 risk model

## Abstract

**Simple Summary:**

Whether it is for COVID-19 primary vaccination or the administration of booster vaccines, prioritization criteria need to be established to optimize COVID-19 vaccination programs accounting for both clinical and social vulnerability risks for severe COVID-19 disease. We developed a dual socio-clinical risk model for severe COVID-19 disease in the Medicare population, which is comprised mostly of individuals aged 65 and over. Our model generated risk levels correlated with regionalized COVID-19 case hospitalization rates and mapped them at the county and zip code levels. The model and map can be used by health jurisdictions to reach out to unvaccinated individuals. Our model approach can also be applied to identify Medicare beneficiaries who were in early vaccination groups to be vaccinated to identify those who might maximally benefit from an additional dose of COVID-19 vaccine if and when vaccine immunity wanes.

**Abstract:**

Recommendations for prioritizing COVID-19 vaccination have focused on the elderly at higher risk for severe disease. Existing models for identifying higher-risk individuals lack the needed integration of socio-demographic and clinical risk factors. Using multivariate logistic regression and random forest modeling, we developed a predictive model of severe COVID-19 using clinical data from Medicare claims for 16 million Medicare beneficiaries and socio-economic data from the CDC Social Vulnerability Index. Predicted individual probabilities of COVID-19 hospitalization were then calculated for population risk stratification and vaccine prioritization and mapping. The leading COVID-19 hospitalization risk factors were non-white ethnicity, end-stage renal disease, advanced age, prior hospitalization, leukemia, morbid obesity, chronic kidney disease, lung cancer, chronic liver disease, pulmonary fibrosis or pulmonary hypertension, and chemotherapy. However, previously reported risk factors such as chronic obstructive pulmonary disease and diabetes conferred modest hospitalization risk. Among all social vulnerability factors, residence in a low-income zip code was the only risk factor independently predicting hospitalization. This multifactor risk model and its population risk dashboard can be used to optimize COVID-19 vaccine allocation in the higher-risk Medicare population.

## 1. Introduction

The question of who should get COVID-19 vaccines was first debated by the National Academy of Medicine, the Centers for Disease Control and Prevention, and by epidemiologists and other disease experts worldwide. U.S. recommendations for the first administration of COVID-19 vaccines focused first on vaccinating individuals at risk for severe disease once health care personnel, nursing home residents, and essential workers had been vaccinated [1]. Individuals over age 65, who are almost all enrolled in the Medicare program, have accounted for a disproportionate share of hospitalizations and 79% of total COVID-19-related deaths [2].

The National Academy of Medicine recommended simultaneously prioritizing individuals living in areas with socio-economic conditions associated with disproportionate vulnerability [3,4] by using the Centers for Disease Control and Prevention (CDC, Atlanta, GA, USA) Social Vulnerability Index (SVI) [5] or the COVID-19 Community Vulnerability Index (CCVI) [6]. Complicating any allocation scheme is the fact that risk categories overlap [7]; more than half of the 53 million Americans over age 65 suffer from two or more chronic conditions [8], and many of them also live in socially vulnerable areas.

Any prioritization scheme, following on the Advisory Committee on Immunization Practices [9], must consider clinical, demographic, and social vulnerability risks together. The CDC listing of risk factors for severe COVID-19 [10] was derived from single hospital-based studies with limited sample sizes [11,12], or hospital reporting, both of which lack nationwide representation [13]. The Center for Medicare and Medicaid Services (CMS) monthly Medicare COVID-19 Data Snapshots include national demographic characteristics and prevalence of common chronic conditions among hospitalized fee-for-service (FFS) Medicare beneficiaries, but these lack more detailed clinical and socio-economic data needed to identify or stratify at risk populations [14]. We are not aware of any published analyses that fully support simultaneous prioritization using both clinical and social vulnerability data.

We developed a model to predict COVID-19 hospitalization and death for Medicare beneficiaries using de-identified Medicare claims which are an important national data resource for studies of the COVID-19 pandemic [15,16,17,18] and CDC Social Vulnerability Index (SVI) data [5]. While the initial impetus for the work, developed for the Department of Defense (DoD) Joint Artificial Intelligence Center (JAIC) [19], was to provide logistics support to hospitals overwhelmed by the pandemic, this model can also support operationalization of the National Academy of Medicine (NAM) and CDC recommendations for a COVID-19 vaccination campaign by stratifying the population by risk, and by mapping locations of beneficiaries in different risk strata.

At a time when we have most recently observed a pandemic uptick due to the SARS-CoV-2 delta variant and may see future pandemic waves due to new variants, this model can be applied to reach out to unvaccinated individuals at highest risk for severe disease to encourage them to get vaccinated. This same modeling approach can also be applied to identify vaccinated Medicare beneficiaries who were first prioritized for vaccination and who might most benefit from an additional dose of COVID-19 vaccine if their level of immunity declines over time.

## 2. Materials and Methods

### 2.1. Data Sources

We constructed an observational cohort consisting of all Medicare FFS beneficiaries who since 1 January 2020, either had a COVID-19 test or a COVID-19 diagnosis (identified by ICD-10 code U071 after 1 April), or for any medical reason were hospitalized or had an emergency department, urgent care, or telehealth visit. The report is based on weekly data outputs from the Center for Medicare and Medicaid Services (CMS, Baltimore, MD, USA) Chronic Condition Warehouse from 1 October 2019 through 22 November 2020, resulting in a weekly download of over 100 million individual records to a secure government enclave of the DoD JAIC for Project Salus’ partner, Humetrix, to process and analyze.

### 2.2. Dependent and Independent Variables

Dependent variables were confirmed COVID-19 cases and their related hospitalizations and deaths. Independent variables included beneficiary age, sex, ethnicity, insurance coverage, the number of prior hospitalizations, comorbidities, dispensed medications and local socio-economic variables from the CDC Social Vulnerability Index. We used the number of prior hospital admissions since 1 October 2019 as an indicator of frailty, and we assessed comorbidities by examining an individual’s diagnoses listed in claims data beginning 1 October 2019. To identify comorbidities, we used a set of chronic conditions flagged by CMS and compiled diagnostic categories using specific ICD-10 code algorithms to identify additional chronic conditions. Medication NDC codes (U.S. Food and Drug Administration, Silver Spring, MD, USA) were used to identify active pharmaceutical ingredients, which were grouped by pharmaceutical class by mapping to RxNorm codes (U.S. National Library of Medicine, Rockville, MD, USA). Socio-economic variables (e.g., income, housing, and other factors) were defined at the individual residential zip code level after conversion from the census track based data found in the CDC Social Vulnerability Index using HUD-USPS [20] crosswalk files (U.S. Department of Housing and Urban Development, Washington, DC, USA). A complete list of independent variables is found in the Supplementary Materials.

### 2.3. Statistical Analysis, Variable Selection, and Risk Model

We used logistic regression to identify significant predictors of COVID-19-related hospitalization or COVID-19 deaths, using R statistical software, version 3.6 with rms, glmnet and pROC packages [21,22,23,24] and R Studio (Boston, MA, USA) using the following binary outcomes: those who received outpatient care only (defined as cases that did not require hospitalization or did not die at least thirty days after diagnosis) versus either those who were hospitalized for COVID-19, or those beneficiaries whose deaths were attributed to COVID-19 (defined as cases who died of SARS-CoV-2 infection within 60 days of diagnosis). We divided the sample into training and validation sets to develop our final models, randomly allocating cases to training (60%) or validation (40%) components, and randomly allocating controls to training or validation components such that the case control ratio is 50:50 in the training set and 45:55 in the validation set (the ratio of COVID-19 hospitalizations to COVID-19 cases we observed in the whole cohort). We examined correlation coefficients between independent variables and used lasso regression to eliminate correlated or collinear independent variables. We then used stepwise backward variable selection procedure based on the Akaike Information Criterion (AIC) to remove non-significant variables.

Because the odds ratios derived from logistic regression models are a measure of the association between a given feature (e.g., North American Native ethnicity) and the outcome (e.g., hospitalization), we supplemented our analyses with a random forest machine learning algorithm, which produces computed Feature Importance values (Python, scikit-learn version 0.22.1 with RandomForestClassifier and GridSearchCV packages) [25] and provides information about the relative importance of each feature for predicting outcomes for the entire sample. As interactions between features are intrinsically built into the Decision Tree structure of the random forest model (but not the Logistic Regression model), the Feature Importance values reflect the relationship between the feature and the outcome even if the relationship depends on the state of another feature in the model. We calculated the Gini importance of each feature in the models to determine which variables were the most important for determining severe disease outcomes in our sample. Trees were built using bootstrapping with balanced subsamples. Parameters specifying the maximum depth of the tree and the number of estimators (trees in the forest) were optimized by cross-validated grid-search in the training set. The data sampling procedure, variable definition, feature engineering, and patient outcome definitions were identical to those described above for Logistic Regression. Statistical analysis details are provided in the Supplementary Materials.

### 2.4. Population-Level COVID-19 Hospitalization Risk Mapping

We used the results of our logistic regression to compute individual predicted probabilities of hospitalization in the event of SARS-CoV-2 infection for the entire analytic cohort of 16 million beneficiaries. We then computed the percentage of the cohort population with a predicted probability of hospitalization of 0.55 or higher, mapping the data at county and zip code levels for the entire country on a nationwide digital dashboard. The predicted probability threshold of 0.55 was selected as it generated higher Pearson’s R values than a threshold of 0.50 in the linear regression analysis of COVID-19 case hospitalization rates as a function of the population risk level in each zip code in six major metropolitan areas shown (see Figure S1 for linear regressions). A sensitivity analysis varying the threshold from 0.40 to 0.65 in steps of 0.05 produced less than a 10% change in Pearson’s R for any of these six metropolitan areas.

## 3. Results

### 3.1. Study Population Characteristics

Socio-demographic and clinical characteristics of the study population (16 million beneficiaries with 1,030,893 confirmed COVID-19 cases, as of 22 November 2020) are summarized in Table 1.

Among COVID-19 cases, patients with the most severe disease resulting in either hospitalization or death had higher frequencies of diabetes, COPD, end-stage renal disease (ESRD), chronic kidney disease, hypertension, ischemic heart disease, cerebrovascular disease, pulmonary fibrosis or pulmonary hypertension, chronic liver disease, asthma and CHF (congestive heart failure) (by Chi Squared tests, all *p* values < 0.001; see Table 1). Patients hospitalized for COVID-19 had similar comorbidity frequencies to those reported by CMS in its monthly Medicare COVID-19 Data Snapshot [14]. With regard to social vulnerability, beneficiaries who were either hospitalized or died due to COVID-19 had higher frequencies of living in zip codes with the lowest income, or highest multiunit housing (by Chi Squared tests, all *p* values < 0.001), or with the most crowded housing (Chi squared tests, all *p* values < 0.01; see Table 1) than patients with less severe disease or beneficiaries who did not have COVID-19.

### 3.2. Individual Predictors

**Figure 1:**
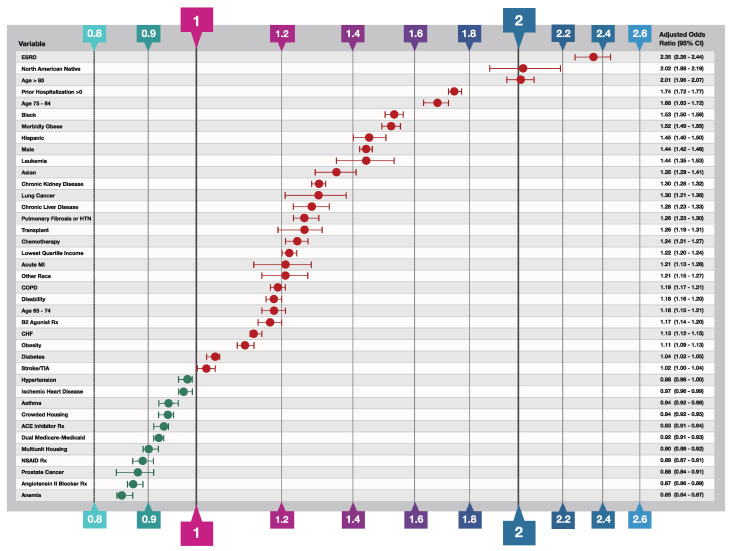
Predictor Variables for COVID-19-Related Hospitalization. The independent variable odds ratios were determined by binary logistic regression analysis of confirmed COVID-19 cases that required hospitalization for the disease versus those that were managed with outpatient care only. In addition to the thirty-nine variables shown in the figure, the following variables were included in the model and survived the variable selection procedure described in Methods but are not shown: colorectal cancer (OR 1.07; 95% CI 1.01–1.14), endometrial cancer in the second half of 2019 (OR 1.12; 95% CI 1.00–1.25), other ethnicity (OR 1.19; 95% CI 1.13–1.25), unknown ethnicity (OR 0.96; 95% CI 0.91–1.00), prescriptions overlapping the COVID-19 diagnosis date of Azithromycin (OR 1.15; 95% CI 1.11–1.18), Chloroquine and Hydroxychloroquine drugs (OR 0.96; 95% CI 0.91–1.01), anticoagulant drugs (OR 1.06; 95% CI 1.04–1.08), opioid drugs (OR 1.03; 95% CI 1.01–1.05) and H2 blocker drugs (OR 1.03; 95% CI 0.99–1.06); Variables excluded from the model by the variable selection procedure included a history breast cancer in the second half of 2019, prescriptions for immunosuppressive and corticosteroid drugs overlapping the COVID-19 diagnosis date, hypertension and pneumococcal vaccinations. B2 Agonist Rx signifies treatment with a beta-2 bronchodilator drug; NSAID Rx signifies treatment with a Non-Steroidal Anti-Inflammatory Drug.

**Figure 2:**
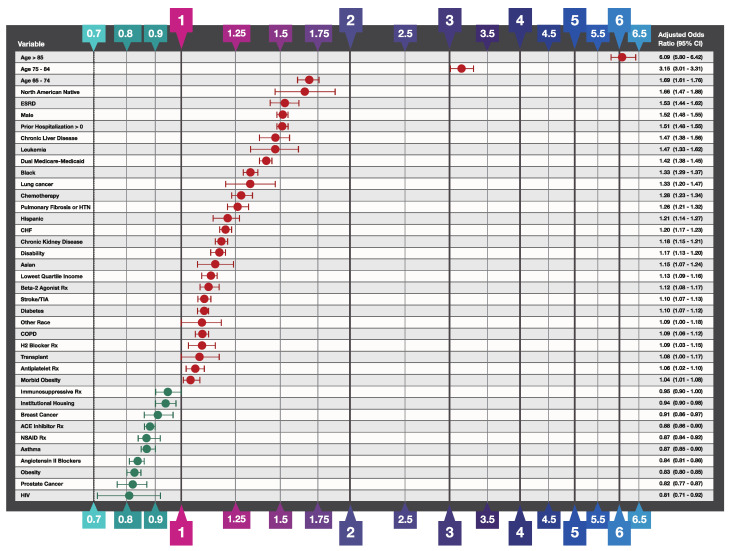
Predictor Variables for COVID-19-Related Death. The independent variable odds ratios were determined by binary logistic regression analysis of confirmed COVID-19 cases that survived versus those that died within 60 days of COVID-19 diagnosis. In addition to, the thirty-nine variables shown in the figure, the following variables were included in the model and survived the variable selection procedure described in Methods but are not shown: prescriptions filled with sufficient quantity to overlap the COVID-19 diagnosis date: Azithromycin (OR 1.18; 95% CI 1.13–1.23), chloroquine and hydroxychloroquine drugs (OR 1.22; 95% CI 1.13–1.23), unknown race (OR 0.88; 95% CI 0.80–0.96). Odds ratios for anemia and prescriptions for anticoagulant drugs and corticosteroids had regression coefficient *p* values > 0.05 and are not shown. Variables excluded from the model by the variable selection procedure include a history of colorectal cancer and endometrial cancer, or acute MI between July and December 2019, ischemic heart disease, hypertension, residence in zip codes in the top quartile of crowded housing or multiunit housing, and prescriptions for opioid drugs. NSAID Rx signifies treatment with a Non-Steroidal Anti-Inflammatory Drug.

Logistic regression adjusted odds ratios identifying predictors of hospitalization and death at the individual level are presented in Figure 1 and Figure 2, respectively. The hospitalization model achieved an Area Under the Receiver Operating Characteristic curve (AUROC) of 0.66 (balanced accuracy 0.61 using threshold 0.50) while the death model achieved an AUROC of 0.71 (balanced accuracy 0.65 using threshold 0.50). Variables excluded from the models based on the specified selection criteria to remove insignificant variables are listed in the legends of Figure 1 and Figure 2.

As expected and as seen in other infectious respiratory diseases such as influenza and pneumonia [26], demographic factors were among the strongest predictors of hospitalization and death. The 85+ age group was twice as likely to be hospitalized relative to the reference level (OR 2.02; 95% CI 1.88–2.19) and six times more likely to die following a COVID-19 diagnosis (death OR 6.09; 95% CI 5.90–6.42). High risk was also observed in other two older age groups: 65–74 (death OR 1.69; 95% CI 1.61–1.76) and 75–85 (hospitalization OR 1.68; 95% CI 1.63–1.72; death OR 3.15; 95% CI 3.01–3.31). North American Natives were twice as likely to be hospitalized (OR 2.02; 95% CI 1.88–2.19) and 66% more likely to die after COVID-19 diagnosis. In addition, Black (hospitalization OR 1.53; 95% CI 1.50–1.56) and Hispanic (OR 1.45; 95% CI 1.40–1.50) ethnicities were associated with higher risk of hospitalization.

Among socio-economic factors extracted from SVI data, the strongest predictor for severe COVID-19 was living in a zip code with the lowest quartile of income (hospitalization OR 1.22; 95% CI 1.20–1.24; death OR 1.13; 95% CI 1.09–1.16). In contrast to income, neither crowded housing nor living in multiunit housing were independent risk factors for either hospitalization or death from COVID-19 (both ORs < 1.0). The socio-economic variables for poverty and education, the housing and transportation summary SVI ranking variable, and the overall summary SVI variable were highly correlated with either income or housing variables and thus excluded from the final models (see Supplemental Materials for more details).

On the clinical side, the strongest risk factors for COVID-19 hospitalization and death were end-stage renal disease (ESRD, hospitalization OR 2.35; 95% CI 2.26–2.44; death OR 1.53; 95% CI 1.44–1.62) and frailty as assessed by one or more prior hospitalization(s) since October 2019 (hospitalization OR 1.74; 95% CI 1.72–1.77; death OR 1.51; 95% CI 1.48–1.55). Morbid obesity and leukemia were leading risk factors for hospitalization; in addition, patients with chronic kidney disease, lung cancer, chronic liver disease, pulmonary fibrosis or pulmonary hypertension, or were undergoing chemotherapy had 20–30% higher risk of hospitalization (see Figure 1 for details). Beneficiaries with leukemia (OR 1.47; 95% CI 1.33–1.62) and chronic liver disease (OR 1.47; 95% CI 1.38–1.56) were associated with a 47% higher risk of death following COVID-19 diagnosis. COPD alone was associated with a modest increased risk of hospitalization (OR 1.19; 95% CI 1.17–1.21). However, beneficiaries with COPD who also had a drug claim for a beta-2 agonist bronchodilator (B2 agonist Rx in Table 1) would have an OR of hospitalization of 1.39 (calculated by adding their respective regression coefficients together) which would put them in the highest risk quartile. On the other hand, diabetes was not a major predictor of hospitalization (OR 1.04; 95% CI 1.02–1.05) but diabetic COVID-19 patients were at a slightly higher risk of death (OR 1.10; 95% CI 1.07–1.12). Contrary to recognized risks for influenza or pneumonia-related hospitalization and death [27] hypertension and asthma were not associated with higher odds of COVID-19 hospitalizations or death. Use of ACE inhibitors (OR 0.93 95% CI 0.91–0.94; OR 0.88; 95% CI 0.86–0.90), angiotensin II blockers (OR 0.87 95% CI 0.86–0.89; OR 0.84 95% CI 0.81–0.86), and Non-Steroidal Anti-Inflammatory Drugs (NSAIDs) (OR 0.89 95% CI 0.87–0.91; OR 0.87 95% CI 0.84–0.92) were modestly associated with lower rates of COVID-19-related hospitalization or death, respectively.

**Figure 3:**
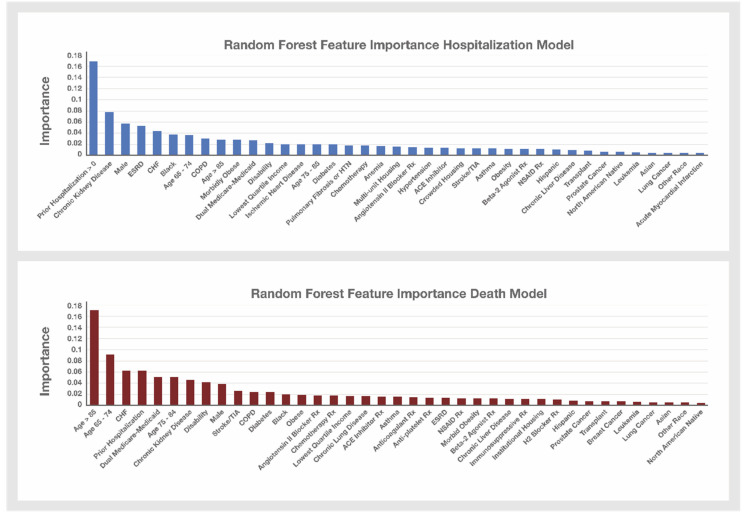
Random Forest Hospitalization and Death Model Feature Importance. Variables that were selected for inclusion in the Hospitalization and Death logistic regression models were used to build these two random forest models. The Feature Importance values for the variables not shown in the Hospitalization model graph are: prescriptions filled with sufficient quantity to overlap the COVID-19 diagnosis date for Azithromycin (FI 0.0104), Chloroquine and Hydroxychloroquine drugs (FI 0.0056), anticoagulant drugs (FI 0.0129), antiplatelet drugs (FI 0.0105), corticosteroids (FI 0.0118), and immunosuppressive drugs (FI 0.100); endometrial cancer (FI 0.002) or breast cancer (FI 0.006) between July and December 2019; unknown race (FI 0.0039) and HIV (FI 0.0045). The Feature Importance values for the variables not shown in the Death model graph include: prescriptions filled with sufficient quantity to overlap the COVID-19 diagnosis date for Azithromycin (FI 0.0107), Chloroquine and Hydroxychloroquine drugs (FI 0.0065), corticosteroids (FI 0.0134), anemia (FI 0.0189), unknown race (FI 0.004) and HIV (FI 0.0037).

The random forest results identifying important factors for predicting hospitalization and death in the Medicare beneficiary sample are presented in Figure 3. The random forest hospitalization model achieved an AUROC of 0.67 while the death model achieved an AUROC of 0.71. The Feature Importance (FI) values for the random forest models are shown in Figure 3, and mainly confirm the regression analysis findings. A history of prior hospitalizations before the diagnosis of COVID-19 was the most important variable in the hospitalization model (FI 0.17), but chronic kidney disease (FI 0.08) and ESRD (FI 0.05) were also identified as important features. The most important predictor of death was age >85 (FI 0.16), but a history of prior hospitalizations (FI 0.06) and congestive heart failure (0.06) were also important.

### 3.3. Population Risk Mapping and Stratification

We used the logistic regression results to calculate a predicted probability of hospitalization for every individual in the cohort based on their individual characteristics and applied these probabilities to map population-level risk nationwide at both zip code and county levels (see Figure S2 in the Supplemental File). These risk maps display, with use of a color gradient, the percentage of our cohort with a predicted probability of hospitalization greater than 0.55 for every residential zip code and county in the U.S.

**Figure 4:**
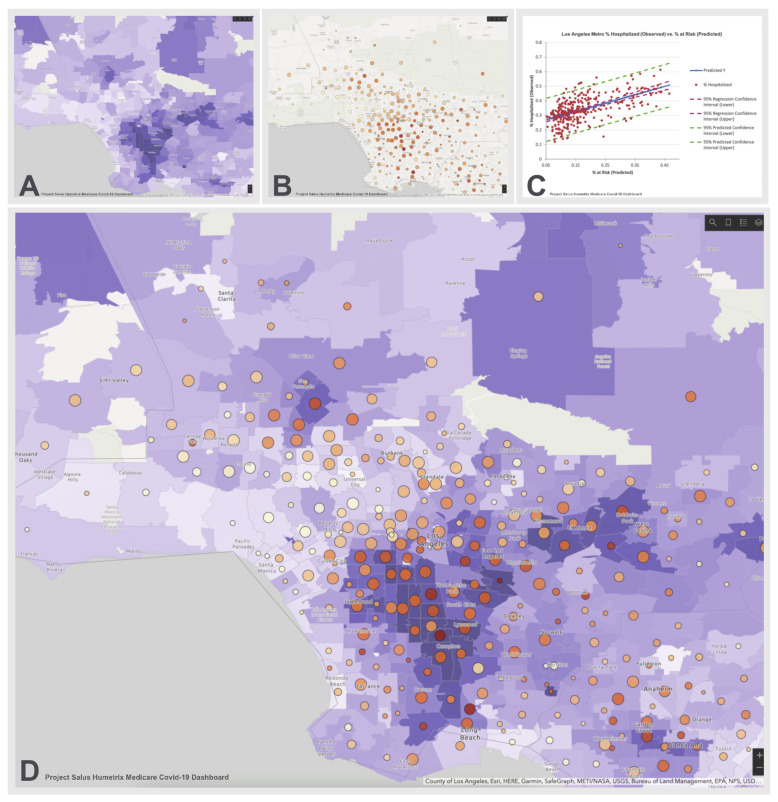
Los Angeles COVID-19 Hospitalization Risk Map. Panel **A** shows the percentage of the Salus cohort with a predicted probability of hospitalization when diagnosed with COVID-19 of over 0.55 on a light blue to dark lavender color scale. Panel **B** shows the cumulative number of hospitalizations per zip code (increasing size of circles denotes a higher hospitalization count) with the percentage of cases requiring hospitalization shown on a beige to dark orange scale. Panel **C** shows a linear regression analysis of the case hospitalization rate (*Y* axis) as a function of the risk level in each zip code (regression R^2^ = 0.35); Panel **D** is an overlay of panel **B** on Panel **A** and demonstrates that zip codes with the highest predicted probabilities of hospitalization with COVID-19 tend to have higher observed percentage of cases requiring hospitalization and vice versa.

Figure 4 displays an example of the risk map in the Los Angeles metropolitan area. In this example, the Los Angeles zip codes displaying a wide range of population percentages over the 0.55 predicted hospitalization probability threshold, were positively correlated with the cumulative COVID-19 case hospitalization rates in these zip codes (Pearson’s R = 0.59, (df = 306; *p* < 0.0001)). We conducted the same correlation and linear regression analyses in 5 other major metropolitan areas in the U.S. All correlation coefficients in these regions had *p* values < 0.0001 by Chi Square. (see Figure S1 in the Supplemental File). For the 15 most populous U.S. metropolitan areas, we also observed a positive correlation of risk levels with COVID-19 case hospitalization rates (mean Pearson’s R = 0.51; 95% CI 0.45–0.56 with *p* values ranging between < 0.0001 and 0.03).

## 4. Discussion

The severe COVID-19 risk models and the mapping of Medicare beneficiaries at highest risk are based, to our knowledge, on the largest COVID-19 dataset assembled to date for this purpose. Importantly, this cohort captured confirmed COVID-19 cases after 1 March 2020, their hospitalizations, and deaths amongst the total 37 million beneficiaries comprising the Medicare FFS population. All members of the study cohort were active users of health care services, presenting on average a frailer clinical profile than the general Medicare population [28], as shown by their clinical characteristics in Table 1. While our observed 1,030,893 Medicare FFS cases represent a little less than 10% of the total number of COVID-19 cases in the U.S., they contributed close to 50% of all CDC estimated COVID-19-related hospitalizations and most Medicare hospitalizations [29]. The scale of this dataset has allowed us to validate our hospitalization model predictions quantitatively and qualitatively with actual COVID-19 case hospitalization rates in multiple geographic areas.

The nationwide distribution of the population also enabled DoD’s Project Salus to produce the first county and zip code level mapping of the population at higher risk for severe COVID-19 and related hospitalizations, with the original goal of providing logistics support to hospitals predicted to experience a surge in patients. Beyond its use for pandemic mapping, the model and related mapping provide information that local health authorities can use to identify zip codes with the highest percentages of FFS beneficiaries at risk of severe COVID-19 who should be prioritized for the administration of primary series or booster doses of COVID-19 vaccines. The same regions should also be a focus of public health efforts to motivate wearing face masks in high-risk indoor settings to reduce the risk of exposure to a large infectious dose of SARS-CoV-2 sufficient to cause COVID-19 infection.

Our combined use of logistic regression and random forest analyses, as applied to this very large Medicare population, allowed us to further assess the current CDC listing of clinical and socio-demographic risk factors for severe COVID-19 [30,31]. Logistic regression and random forest analyses provide two different perspectives: the odds ratios from the logistic regression help identify individual risk while the Feature Importance from the random forest analyses identified the most important variables for predicting severe COVID-19 outcomes at the population-level for the entire cohort. Secondly, Feature Importance values reflect the relationship between the Feature and the outcome, including where the relationship depends on the state of another Feature in the model.

Unlike odds ratios which are not influenced by the prevalence of the Feature in the population, Feature Importance is also more influenced by how common a Feature is within the sample. Thus, while North American Native ethnicity has one of the highest odds ratios in the hospitalization logistic regression model, its Feature Importance in the random forest model was low (ranked 42nd out of 50 variables) due to the low frequency of this feature in the sample (<1% of the sample). The random forest analysis identifies CHF, COPD and chronic kidney disease which, unlike North American Native ethnicity, are highly prevalent in this cohort (cohort prevalence: 24.4%, 25.8%, 36.7%, respectively) as among the most important risks for hospitalization (ranked #5, #8 and #2, respectively) and death (ranked #3, #11 and #7, respectively) from COVID-19 for the entire cohort, even though each of these conditions at the individual level are associated with modest risks with odds ratios all under 1.3 in the logistic regression hospitalization and death models.

Both logistic regression and random forest analyses affirm the critical risk factors of ethnicity [32], older age, and morbid obesity [33] as previously reported. Both logistic regression and random forest models also show that a prior hospitalization, a marker of frailty in aged Medicare beneficiaries [34], was one of the most significant individual characteristics associated with severe COVID-19 outcomes.

However, contrary to prior descriptive analyses performed on smaller population sizes of hospitalized patients only [35,36,37], or to a recently published report identifying risk of hospitalization associated with chronic conditions not adjusted for age, ethnicity or prior hospitalization in the fee-for-service Medicare population [15] our study reveals the lack of, or modest effect of, hypertension, diabetes, COPD, and asthma in the hospitalization and death logistic regression models. Our logistic regression results confirms the finding in this report that ESRD is the strongest COVID-19 hospitalization risk factor, and affirms North American Natives have the highest risk of hospitalization among ethnic minorities. Furthermore, in the national risk map (Supplemental File Figure S2) based on our logistic regression hospitalization model, 26 counties with an average percent North American Native population of 68% have among the highest percentage of individuals at high risk of severe COVID-19 in the United States.

One of the main limitations of our study and derived models is that it is only based on the Medicare FFS population, which represents approximately 60% of the total Medicare population, with regional variations ranging from 98% in Alaska to 51% in Minnesota. There is evidence that Medicare Advantage plans tend to enroll beneficiaries who are healthier than Medicare FFS beneficiaries, this difference in health status will limit the generalization of our model to the entire Medicare population. If the model were to be used for vaccine allocation, it could be updated using Medicare Advantage data if these were made available. Finally, fifteen percent of this FFS Medicare study cohort are beneficiaries under the age of 65 who are either disabled or have end-stage renal disease (ESRD) or amyotrophic lateral sclerosis which limits the applicability of our findings to the over 65 U.S. population.

**Figure 5:**
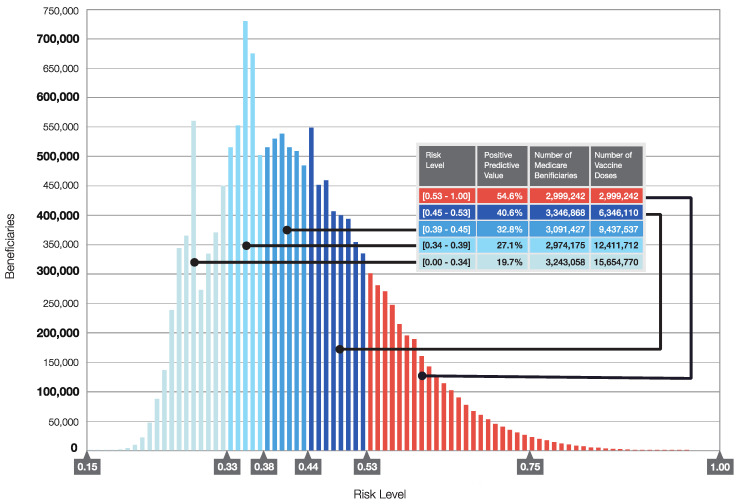
COVID-19 Vaccine Prioritization Based on Risk of Severe COVID-19 Disease. The logistic regression model coefficients for the independent variables shown in Figure 1 were used to calculate the predicted probabilities of hospitalization in the Salus cohort. The distribution of predicted probabilities was split into five groups shown in the table of approximately 3 million beneficiaries each to enable stratification of the cohort by risk of severe disease to prioritize individuals for COVID-19 vaccination.

With the above limitations, the models we have developed provide important information for public health officials and advisory groups who recommend COVID-19 vaccination and other preventive behaviors to consider. Specifically, because the models integrating both socio-economic factors and individual clinical data respond to the recommendations of the NAM for prioritization and allocation of COVID-19 vaccines, they could be used to support planning a vaccination campaign. Figure 5 displays a histogram of the distribution of the predicted probabilities of hospitalization for SARS-CoV-2 infected patients, and when such data are mapped, they enable planners to estimate how many high-risk beneficiaries reside in a jurisdiction, and of those, how many are in socially vulnerable areas. Our models identifying individuals at risk for severe COVID-19 could also be used by the Medicare program, in collaboration with state and local health officials, to provide incentives for completion of COVID-19 vaccination and adherence to protective behaviors including wearing facemasks in settings with high risk of exposure to large doses of SARS-CoV-2 in aerosols. Additionally, as is done for electricity dependent persons in other emergencies, local health officials can request names and addresses of these beneficiaries in their jurisdictions, provided they can HIPAA-protect the data, and then could conduct direct outreach to beneficiaries at highest risk. Further, once receipt of vaccination is linked to Medicare claims, the analytic platform could be used to support post-licensure pharmacovigilance and effectiveness studies, especially for those identified at higher risk of severe COVID-19. These are of paramount importance, especially in the early phases of a vaccination campaign.

## 5. Conclusions

This quantitative analysis of risk for severe COVID-19 in the Medicare population provides important new insights useful for managing the COVID-19 vaccination campaign. At this time of unpredictable pandemic upticks due to new or evolving SARS-CoV-2 variants, this risk model can be applied to reach out in priority to unvaccinated individuals at highest risk of severe disease, either by area or using individual identifiers. Our risk model development approach can also be applied when considering booster administration if waning vaccine immunity takes place starting with individuals at higher risk [38]. To be actionable, state and local governments could consider asking the DoD, through the usual Mission Assignment process used to provide domestic support, to provide the risk mapping to their jurisdictions. Finally, this work underscores the value of Medicare claim data for epidemiologic surveillance, which with its size and nationwide representation can also augment both the ILINet [39] and COVID-NET [40] disease surveillance systems and complement the existing vaccine monitoring systems for tracking both the safety and efficacy of COVID-19 vaccination in the high-risk Medicare population [18].

## Figures and Tables

**Table 1:** Demographic, Clinical and Socioeconomic Characteristics of the Project Salus Medicare Cohort.

Variable		Non-Covid-19 Cases †		Covid-19 Outpatients ‡		Covid-19 Hospitalized ¶		Covid-19 Deaths #	
	Total	14,592,352		421,575		345,111		135,567	
Age		Median	(IQR)	Median	(IQR)	Median	(IQR)	Median	(IQR)
	Age	73	(67–80) ^✦✦✦^	73	(68–81)	76	(68–84) ^✦✦✦^	82	(73–89) ^✦✦✦^
		%	(no.)	%	(no.)	%	(no.)	%	(no.)
	Under 65	15.20%	(2,221,832) ***	14.60%	(61,446)	14.30%	(49,202) ***	7.40%	(10,062) ***
	From 65 to 74	41.50%	(6,061,460) ***	40.40%	(170,315)	32.10%	(110,873) ***	21.50%	(29,161) ***
	From 75 to 84	28.80%	(4,208,784) ***	27.00%	(113,939)	30.80%	(106,170) ***	31.20%	(42,248) ***
	Over 85	14.40%	(2,100,276) ***	18.00%	(75,875)	22.90%	(78,866) ***	39.90%	(54,096) ***
Sex		%	(no.)	%	(no.)	%	(no.)	%	(no.)
	Male	43.40%	(6,330,698)	40.50%	(170,855)	48.50%	(167,259) ***	48.40%	(65,679) ***
	Female	56.60%	(8,261,652)	59.50%	(250,720)	51.50%	(177,852) ***	51.60%	(69,888) ***
Race		%	(no.)	%	(no.)	%	(no.)	%	(no.)
	North American Native	0.60%	(90,876) ***	0.50%	(2285)	1.00%	(3410) ***	0.90%	(1171) ***
	Black	9.60%	(1,394,033) ***	12.50%	(52,738)	18.10%	(62,302) ***	16.50%	(22,338) ***
	Hispanic	2.00%	(293,861) ***	3.80%	(15,971)	4.70%	(16,125) ***	4.20%	(5729) ***
	Asian	1.90%	(277,030) ***	2.10%	(8728)	2.30%	(7857) ***	2.40%	(3248) ***
	White	82.20%	(12,002,137) ***	77.20%	(325,301)	70.80%	(244,233) ***	73.40%	(99,458) ***
	Other	1.60%	(227,572) *	1.70%	(7057)	1.70%	(5890)	1.60%	(2223)
	Unknown	2.10%	(306,843) ***	2.30%	(9495)	1.50%	(5294) ***	1.00%	(1400) ***
Socio-economic (SVI) Variable Quartiles		%	(no.)	%	(no.)	%	(no.)	%	(no.)
	Lowest Income (EPL_PCI)	11.20%	(1,635,128) ***	13.70%	(57,813)	17.60%	(60,773) ***	16.80%	(22,732) ***
	Most Crowded Housing (EPL_CROWD)	10.00%	(1,452,010) ***	16.20%	(68,406)	16.50%	(56,957) **	16.60%	(22,475) ***
	Highest Multi-unit Housing (EPL_MUNIT)	11.70%	(1,700,037) ***	17.70%	(74,648)	16.00%	(55,163) ***	16.70%	(22,659) ***
	Highest Institutional Housing (EPL_GROUPQ)	7.50%	(1,092,374)	7.10%	(29,776)	7.00%	(24,092)	7.20%	(9731) ***
		%	(no.)	%	(no.)	%	(no.)	%	(no.)
Disabled		24.80%	(3,619,734) ***	26.00%	(109,502)	27.40%	(94,554) ***	21.80%	(29,522) ***
Dual Medicare-Medicaid		21.80%	(3,187,875) ***	37.50%	(158,292)	40.00%	(137,894) ***	47.70%	(64,679) ***
Prior Hospitalization		%	(no.)	%	(no.)	%	(no.)	%	(no.)
	0	100.00%	(14,592,352) ***	79.20%	(333,743)	64.20%	(221,694) ***	62.20%	(84,328) ***
	1 or more	0.00%	NA	20.80%	(87,832)	35.80%	(123,417) ***	37.80%	(51,239) ***
Clinical Variables		%	(no.)	%	(no.)	%	(no.)	%	(no.)
	ESRD	1.80%	(260,810) ***	2.10%	(8940)	6.30%	(21,703) ***	5.00%	(6814) ***
	Chronic Kidney Disease	36.70%	(5,351,947) ***	39.50%	(166,536)	53.00%	(182,910) ***	57.60%	(78,103) ***
	Pulmonary Fibrosis or HTN	7.10%	(1,030,957) ***	5.50%	(23,231)	9.00%	(31,198) ***	9.40%	(12,677) ***
	Chronic Liver Disease	2.60%	(379,804) ***	2.50%	(10,525)	3.80%	(13,032) ***	3.60%	(4901) ***
	COPD	25.80%	(3,764,077) ***	27.70%	(116,650)	36.20%	(124,828) ***	39.70%	(53,790) ***
	CHF	24.40%	(3,553,556) ***	29.70%	(125,223)	40.70%	(140,534) ***	48.20%	(65,366) ***
	Stroke/TIA	13.50%	(1,968,280) ***	17.90%	(75,473)	22.30%	(77,087) ***	28.40%	(38,437) ***
	Diabetes	37.30%	(5,441,056) ***	44.50%	(187,722)	52.70%	(182,045) ***	55.30%	(75,020) ***
	Hypertension	76.10%	(11,107,034) ***	79.10%	(333,506)	84.80%	(292,744) ***	88.70%	(120,301) ***
	Acute MI	0.90%	(130,435) ***	0.80%	(3327)	1.50%	(5101) ***	1.60%	(2160) ***
	Ischemic Heart Disease	44.00%	(6,427,825) ***	48.90%	(206,139)	57.60%	(198,625) ***	64.40%	(87,267) ***
	Asthma	16.20%	(2,360,423) ***	17.50%	(73,567)	19.60%	(67,752) ***	18.70%	(25,301) ***
	Chemotherapy	9.10%	(1,321,542) ***	11.30%	(47,845)	14.40%	(49,598) ***	13.60%	(18,494) ***
	Obesity	15.10%	(2,200,797) ***	16.30%	(68,926)	16.70%	(57,706) ***	11.90%	(16,076) ***
	Morbid Obesity	8.70%	(1,264,568) ***	9.80%	(41,133)	13.90%	(48,083) ***	9.60%	(13,013) ***

† Asterisks shown in this column indicate *p* values for the differences between individuals who have not been diagnosed with Covid-19 and confirmed Covid-19 cases in the Salus Medicare cohort. ‡ Covid-19 outpatients are defined are individuals who did not require hospitalization for the disease and remained alive at least 30 days after diagnosis. Covid-19 cases who either did not die or were not hospitalized for the disease but who had no claims more than 30 days after their Covid-19 diagnosis are not shown in this table. ¶ Covid-19 hospitalized cases are those requiring inpatient admission for management of their disease. Asterisks shown in this column indicate the *p* values for differences between this group and the Covid-19 outpatient group. # Covid-19 deaths are deaths attributed to Covid-19 based on the timing of death in relation to the date of diagnosis. Asterisks shown in this column indicate the *p* values for differences between Covid-19 cases who died from the disease and those who survived. * *p* < 0.05; ** *p* < 0.01; *** *p* < 0.001 by Chi Square test. ^✦✦✦^ *p* < 0.001 by Mann-Whitney test.

## Data Availability

The study data cannot be made available under the terms of the data use agreement between the U.S. Department of Defense and the Centers for Medicare and Medicaid Services.

## Supplementary Materials

The following are available in the online Supplemental File at https://www.mdpi.com/article/10.3390/biology10111185/s1: Supplemental Methods; Supplemental Results: Figure S1. Metropolitan region risk maps for severe COVID-19; Figure S2. Nationwide county COVID-19 hospitalization risk map

## Department of Defense Disclaimer

The views expressed in this article are those of the authors and do not necessarily reflect the official policy or position of the Department of Defense, nor the U.S. Government. Authors Hassan Tetteh and Peter Walker are military service members. This work was prepared as part of their official duties. Title 17 USC§105 provides that copyright protection under this title is not available for any work of the U.S. Government. Title 17 USC§101 defines a U.S. Government work as a work prepared by a military service member or employee of the U.S. Government as part of that person’s official duties.
