# Supplementary material for: A Predictive Model for Severe COVID-19 in the Medicare Population: A Tool for Prioritizing Primary and Booster COVID-19 Vaccination
Source: Biology (Basel). 2021 Nov 15;10(11):1185. doi: 10.3390/biology10111185 (PMC8614832; doi:10.3390/biology10111185)
Supplement: Supplementary file 1 [file biology-10-01185-s001.zip › biology-1432960-supplementary.pdf]

Supplemental File

Table of Contents:

Supplemental Methods 2–6

Medicare claim data processing..2
Logistic regression model training...2
Random forest model training...2
Model validation...3
Demographic and insurance coverage variables..3
Social Vulnerability Index variables..3
CMS chronic condition variables...3
ICD10 codes to identify chronic condition variables.......................................4
Medication variables..5
Covid-19 outcome variables..6
Supplemental Results 7–10

Figure S1...8–10
Figure S2...10

Supplemental Methods:

Medicare claim data processing: The Humetrix SaaS platform installed in the SUNet secure classified government network provides automated pre-processing of weekly updates downloaded to the Humetrix SUNet enclave of Medicare Part A inpatient and outpatient, Hospice and SNF, Part B Carrier claims and Part D (PDE) claims data to generate output files containing derived variables used to train logistic regression and random forest models as described below. The platform's medical terminology service includes a complete collection of AMA CPT-4, FDA NDC, NNPES NPI, ICD-10-CM, CMS Level II HCPCS, and NLM RxNorm codes and provides automated identification grouping of ICD-10-CM codes to identify chronic condition categories and NDC drug code to RxNorm ingredient code mappings to identify pharmaceutical classes of active pharmaceutical ingredients from Medicare claim data.

Logistic Regression Model Training: We used logistic regression to identify significant predictors of Covid-19 related hospitalization or Covid-19 deaths, (R statistical software, version 3.6 with rms, glmnet and pROC packages) using the binary outcomes of those who received outpatient care only (defined as cases that did not require hospitalization or didn't die at least thirty days after diagnosis) versus either those who were hospitalized for Covid-19, or those beneficiaries whose deaths were attributed to Covid-19 (defined as cases who died of SARS-CoV-2 infection within 60 days of diagnosis).

We divided the sample into training and validation sets to develop our final models, randomly allocating cases to training (60%) or validation (40%) components, and randomly allocating controls to training or validation components so that the cases:controls ratio is 50:50 in the training set and 45:55 in the validation set (the ratio we observe in the whole cohort). We examined correlation coefficients between independent variables, and used lasso regression, to eliminate correlated or collinear independent variables. We then used stepwise backward variable selection procedure based on the Akaike Information Criterion (AIC) to remove non-significant variables. Computation of the 95% confidence intervals for the coefficient estimates were generated by bootstrapping (2000 repetitions) on the training set.

Random Forest Model Training: We computed Feature Importance (Python, scikit-learn version 0.22.1 with RandomForestClassifier and GridSearchCV packages). Trees were build using bootstrapping with balanced subsamples. Parameters specifying the maximum depth of the tree and the number of estimators (trees in the forest) were optimized by cross-validated grid-search in the training set. The data sampling procedure, variable definition, feature engineering, and patient outcome definitions were identical to those described above for Logistic Regression.

The Random Forests algorithm is an ensemble method that learns to predict the outcome of a patient by training multiple Decision Tree classifiers. For each Decision Tree in the Random Forest, randomness is introduced both by selecting a random subset of the dataset using the bagging method (sampling performed with replacement) and by searching among a random subset of features for the best feature to split a decision node. The Random Forest then aggregates the outcome predictions from all of the Decision Trees to predict the most frequently predicted outcome (via majority vote).

Decision tree classifiers are greedy algorithms that search for the feature and threshold that will divide the data set into the purest subsets (called child nodes). The training algorithm then selects another feature and threshold combination to further subdivide the child node. This process continues until a stopping condition is reached. The purity of a subset (or node in the Decision Tree) is measured using the Gini Impurity:

$$G_i = 1 - \sum_{j=1}^{N} {p_{i,j}}^2$$

where $p_{i,j}$ is the ratio of the number of observations of a given outcome j among the total number of observations of any outcome in the subset at the ith node.

Random Forest feature importance measures the relative importance of each feature by computing the reduction in impurity attributed to splitting decision nodes using the

feature (weighted by the number of observations in the decision node and averaged across all Decision Trees in the Random Forest).

Model Validation: Model performance for both logistic regression and random forest hospitalization models was measured by the Area Under the Receiver Operating Characteristics (AUROC). The AUROC was computed on a validation set composed of cases that were not used in the training set, with adjustments to give a 55:45 ratio of outpatient to hospitalized cases (which is the observed ratio for hospitalization in the Covid-19 study population). For the logistic regression and random forest death model the AUROC was computed using an 85:15 ratio (which is the observed case fatality ratio in the Covid-19 study population).

Demographic and Insurance Coverage Variables: weekly updates of CMS "Master Beneficiary Summary 2020 File" (MBSF_2020) data were processed, extracting variables: ORIG_REASON_FOR_ENTITLEMENT (Disability), ZIP_CD (residential zip code), YOB, SEX_CODE, RACE_CODE, ESRD_INDICATOR and DUAL_STUS_CD 01-12 (Dual Medicare-Medicaid insurance).

Social Vulnerability Index (SVI) variables associated with zip codes: these were derived from CDC data, which are categorized by census tract. The variables analyzed using binary logistic regression to determine significant predictor variables for hospitalization or death due to Covid-19 included the following SVI variables: the EPL_PCI income variable, the EPL_CROWD (crowded living), EPL_GROUPQ (institutional housing), and EPL_MUNIT (living in multiunit housing); EPL_POV, EPL_NOHSDP (no high school diploma), RPL_THEME1 (summary socioeconomic variable) were all highly correlated with EPL_PCI and excluded on this basis from model training. RPL_THEME4 was excluded based on its correlations with the housing variables EPL_GROUPQ, EPL_CROWD and EPL_MUNIT as well as not surviving AIC based stepwise regression in prior modeling work; RPL_THEMES (the overall tract summary ranking variable) was excluded based on its high correlation with the low-income variable EPL_PCI.

CMS chronic condition variables: These chronic condition codes found in the MBSF_2019 chronic condition segment file were used in hospitalization and death models:

1. Acute myocardial infarction July-December 2019 (AMI)
2. Chronic kidney disease (CHRONIC_KIDNEY_EVER), COPD (COPD_EVER)
3. Congestive heart failure (CHF_EVER)
4. Diabetes mellitus (DIABETES_EVER)
5. Ischemic heart disease (ISCHEMICHEART_EVER)
6. Stroke/transient ischemic attack (STROKE_TIA_EVER)
7. Breast cancer July-December 2019 (CANCER_BREAST)
8. Colorectal cancer July-December 2019 (CANCER_COLORECTAL)
9. Prostate cancer July-December 2019 (CANCER_PROSTATE)
10. Lung cancer July-December 2019 (CANCER_LUNG)
11. Endometrial cancer July-December 2019 (CANCER_ENDOMETRIAL)
12. Anemia July-December 2019 (ANEMIA)
13. Asthma (ASTHMA_EVER)
14. Hypertension (HYPERT_EVER)

ICD10 codes to identify chonic condition variables (present prior to the Covid-19 diagnosis date). The following chronic condition variables were identified by identifying ICD-10-CM codes for the conditions listed below in these CMS claim files: Part A institutional Inpatient and Outpatient claims, Part B Carrier claims, SNF and Hospice claims from October 1, 2019 received by CMS Chronic Condition Warehouse by November 11, 2020.

Leukemia (includes acute and chronic myeloid and lymphocytic leukemias as well as less common leukemias); ICD-10-CM codes:

C95, C959, C9590, C9592, C94, C950, C9500, C9502, C92, C929, C9290, C9292, C951, C9510, C9512, C91, C919, C9190, C9192, C93, C939, C9390, C9392, C943, C9430, C9432, C914, C9140, C9142, C901, C9010, C9012, C91A, C91A0, C91A2, C926, C9260, C9262,

C92A, C92A0, C92A2, C92Z, C92Z0, C92Z2, C913, C9130, C9132, C916, C9160, C9162, C91Z, C91Z0, C91Z2, C921, C9210, C9212, C93Z, C93Z0, C93Z2, C940, C9400, C9402, C948, C9480, C9482, Z806, C920, C9200, C9202, C910, C9100, C9102, C911, C9110, C9112, C924, C9240, C9242, Z856, C925, C9250, C9252, C915, C9150, C9152, C931, C9310, C9312, C942, C9420, C9422, C933, C9330, C9332, C922, C9220, C9222, C930, C9300, C9302

Pulmonary fibrosis or pulmonary hypertension (include idiopathic pulmonary fibrosis, interstitial lung disease, pneumoconioses, pulmonary sarcoidosis, pulmonary hypertension); ICD-10-CM codes:

J61, J62, J620, J628, J63, J636, J65, J64, J60, J84, J841, J8417, J848, J849, J8410, J84112, E84, E840, E841, E8419, E848, E849, I270, I272, I2720, I2722, I2723, I2724, D860.

Chronic liver disease (includes alcoholic cirrhosis, primary biliary cirrhosis, chronic viral hepatitis due to hepatitis B and C, alcoholic fatty liver, primary sclerosing cholangitis, Wilson's disease); ICD-10-CM codes:

K7469, K745, K703, K7030, K7031, P7881, K7460, K74, K743, K744, K746, K717, B18, B180, B181, B182, B188, B189, K70, K709, E8301, K8301, K700, E8801

HIV/AIDS; ICD-10-CM codes:

B20, Z717, O9873, O9872, O98719, O98713, O98712, O98711, O9871, O987, B9735, Z21, Z830

Transplant (includes following transplants: lung, bone, heart, liver, pancreas, intestine, kidney, bone marrow); ICD-10-CM codes:

Z94, Z949, Z9885, D47Z1, T86810, T86811, T86812, Z942, Z945, Z946, T8621, T8622, T8623, T8641, T8642, T8643, T86890, T86891, T86892, Z941, Z944, Z948, T8611, T8612, T8613, Z940, T86840, T86841, T86842, Z947, Z9483, T86850, T86851, T86852, Z9482, T8631, T8632, T8633, Z9484, T8601, T8602, T8603, T8691, T8692, T8693, Z9481, Z7682, Z943, T86, T8681, T862, T864, T868, T8689, T861, Y830, T8685, T865, T863, T86818, T869, T860, T8619, T8629, T8649, T86898, T86848, T86819, T86858, T8620, T8639, T8640, T86899, C802, T8609, T8610, T8699, T86849, T86859, T8630, Z4824, T8600, T8690, Z482, Z4821, Z4823, Z4822, I2575, I25750, I25751, I25758, I25759, I25811, Z48280, Z4829, Z48290, Z4828, I2576, I25760, I25761, I25768, I25769, I25812, I257

Obesity (BMI 30 kg/m^2 - 40kg/m^2); ICD-10-CM codes:

Z6830, Z6831, Z6832, Z6833, Z6834, Z6835, Z6836, Z6837, Z6838, Z6839, E6609, E661, E668, E669

Morbid obesity (BMI over 40kg/m^2); ICD-10-CM codes:

Z6841, Z6842, Z6843, Z6844, Z6845, E6601, E662

Medication variables: PDE and Claims Data Files were analyzed by the Humetrix SaaS system to derive the variables listed below. Variables 2-11 were identified by mapping NDC drug product codes to RxNorm ingredient codes belonging to the indicated pharmaceutical classes of drugs. A code of 1 (=True) was only assigned to these variables if a prescription was filled with a sufficient quantity to overlap the date of the first claim with a Covid-19 ICD-10-CM code. For variable 1 (chemotherapy), the same rule applied to PDE claim NDC codes. For variable 12 (Azithromycin and Chloroquine/Hydroxychloroquine), we also assigned a code of 1 for a PDE claim date up to 10 days beyond the first claim with a Covid-19 ICD-10-CM code. RxNorm TTY = IN ingredient codes for all pharmaceutical classes available on request.

1. Chemotherapy: signifies that a beneficiary at any time in 2020 either had an ICD10 code for chemotherapy in part A Institutional or Part B Carrier claims, had a HCPCS or CPT-4 code indicating administration of parenteral chemotherapy in a Part B claim, or had a pharmacy (PDE) claim with an NDC code which mapped to an RxNorm ingredient code for an active pharmaceutical ingredient belonging to multiple classes of chemotherapeutic agents based on the National Cancer Institute list of drugs used in the treatment of cancer (https://www.cancer.gov/about-cancer/treatment/drugs#F). HCPCS, CPT-4, ICD-10-CM codes are shown below.

CPT-4 chemotherapy codes:

96401, 96402, 96405, 96406, 96409, 96411, 96413, 96415, 96416, 96417, 96420, 96422, 96423, 96425, 96440, 96446, 96450, 96542, 96549, G0498

HCPCS chemotherapy codes:
J8501, J8510, J8515, J8520, J8521, J8530, J8560, J8562, J8565, J8600, J8610, J8650, J8700, J8705, J8999, J9000, J9010, J9015, J9017, J9019, J9020, J9022, J9023, J9025, J9027, J9032, J9033, J9034, J9035, J9036, J9039, J9040, J9041, J9042, J9043, J9044, J9045, J9047, J9050, J9055, J9057, J9060, J9065, J9070, J9098, J9100, J9119, J9120, J9130, J9145, J9150, J9151, J9153, J9160, J9171, J9173, J9176, J9178, J9179, J9181, J9185, J9190, J9199, J9200, J9201, J9203, J9204, J9205, J9206, J9207, J9208, J9210, J9211, J9212, J9213, J9214, J9215, J9216, J9228, J9229, J9230, J9245, J9250, J9260, J9261, J9262, J9263, J9264, J9265, J9266, J9267, J9268, J9269, J9270, J9271, J9280, J9285, J9293, J9295, J9299, J9300, J9301, J9302, J9303, J9305, J9306, J9307, J9308, J9309, J9310, J9311, J9312, J9313, J9315, J9320, J9325, J9328, J9330, J9340, J9351, J9352, J9354, J9355, J9356, J9357, J9360, J9370, J9371, J9390, J9395, J9400, J9600, J9999

ICD-10-CM chemotherapy codes:
D61810, Z5112, Z511, D6481, Z5111, Z511, D701, T80810, T80810D, T80810S, T80810A, D701, D702

2. Anticoagulant drugs (VKORC1 and factor X inhibitors)

3. Antiplatelet drugs (cyclooxygenase inhibitors, ADP receptor inhibitors, adenosine reuptake inhibitors, phosphodiesterase inhibitors)

4. Beta-2 agonists

5. Corticosteroids (excludes topical and ophthalmic preparations)

6. Opioid drugs

7. Histamine type-2 receptor blockers

8. Angiotensin converting enzyme inhibitors (ACE inhibitors)

9. Angiotensin II receptor blockers

10. Non-steroidal anti-inflammatory (NSAID) drugs

11. Immunosuppressive drugs of diverse pharmaceutical classes including anti-interleukins, anti-TNFa drugs, JAK kinase inhibitors, anti-interferons, Sphingosine 1-p receptor modulators, calcineurin inhibitors, mycophenolate and sirolimus and methotrexate.

12. Azithromycin and Chloroquine drugs (includes both Chloroquine and Hydroxychloroquine)

<u>Covid-19 outcome variables</u>:

1. Covid-19 hospitalizations were identified either by Part B Carrier claims with place of service code = 21 or CPT codes indicating inpatient care with a date of service no more than 14 days after or 10 days before the Covid-19 diagnosis date, or by finding Part A Inpatient claims where the data of admission no more than 14 days after or 10 days before the Covid-19 diagnosis date.

CPT-4 codes indicating inpatient services
99217, 99218, 99219, 99220, 99221, 99222, 99223, 99224, 99225, 99226, 99231, 99232, 99233, 99234, 99235, 99236, 99238, 99239

2. Covid-19 cases managed by outpatient care only were defined as cases who were not hospitalized in the 30 days after the Covid-19 diagnosis date and who did not die within 60 days of the diagnosis date.

3. Deaths due to Covid-19 were defined as either a death which occurred during a Covid-19 hospitalization using the inpatient PTNT_DSCHRG_STUS_CD code, or a death which occurred within 60 days of the Covid-19 diagnosis as reported in the DEATH_DT field of the MBSF_2020 file.

Supplemental Results:

<u>Effect size calculation (Table 1)</u>: Due to the very large sizes of the compared populations, the p values for the statistical tests presented in Table 1 are almost all very significant. We used Cramér's V to calculate effect sizes values for the comparisons between groups for binary and categorical variables (age ranges, sex, race, income, poverty, dual Medicare-Medicaid dual status, disabled status, prior hospitalization, and clinical variables) all of which had a computed Cramér's V values under 0.17 indicating a small effect size. We used Vargha and Delanay's A (VDA) to calculate effect sizes for comparisons

between groups for the quantitative variable of age in years and found VDA values of 0.45 (patients with Covid-19 versus those without), 0.55 for those hospitalized for Covid-19 versus those managed as outpatients, and 0.66 for those who died from the disease versus survivors all indicative of small effect sizes.

Analysis of Hospitalization model predictions of Covid-19 case hospitalization rates in six large metropolitan areas (see following pages).

Figure S1.

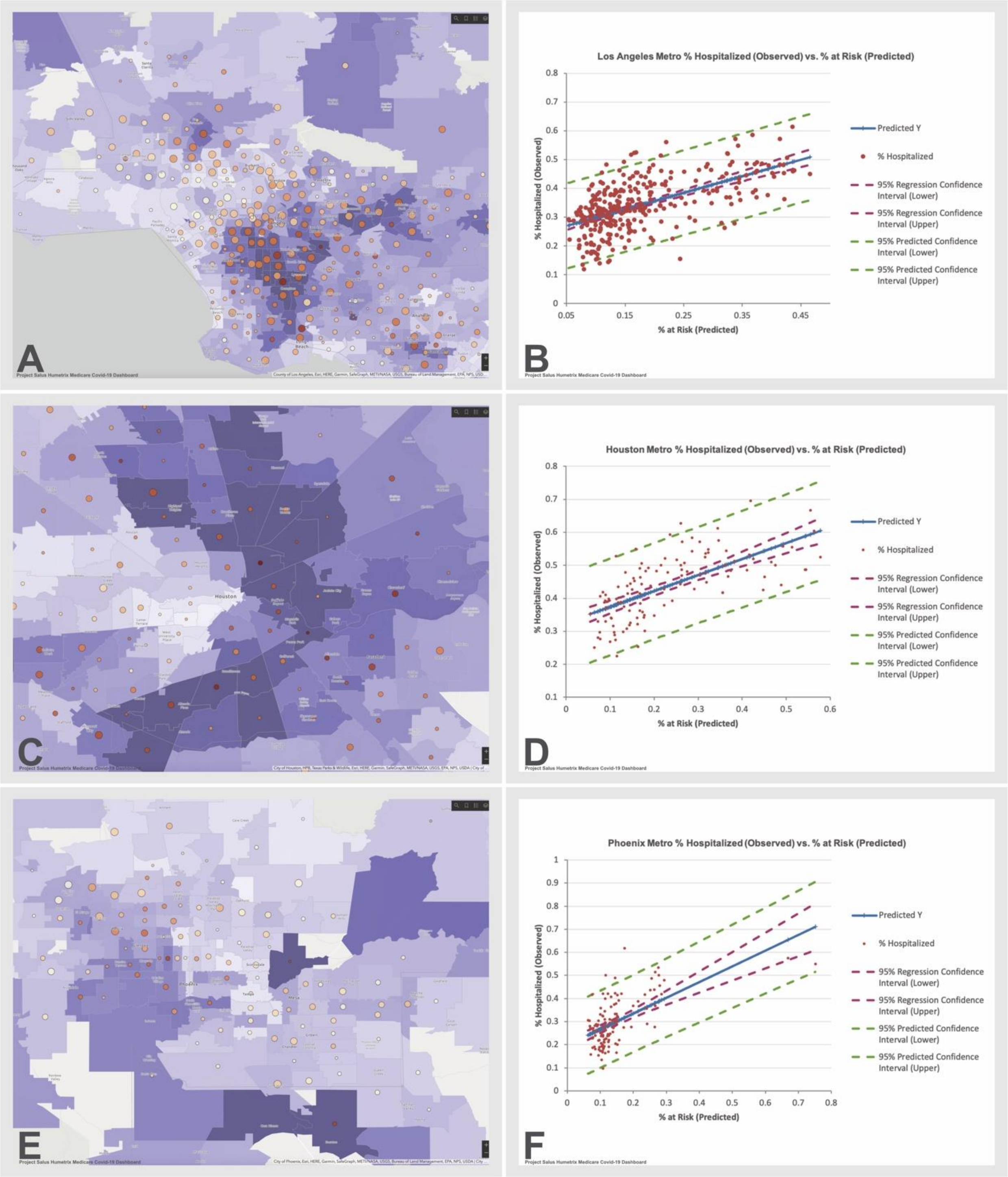

Figure S1. Metropolitan region risk maps for severe Covid-19 (A) Los Angeles County, (C) Houston, (E) Phoenix, (G) New York City, (I) Miami-Dade - Palm Beach, (K) Washington DC. In each metropolitan region, zip codes with the higher predicted probabilities of hospitalization with Covid-19 based on the logistic regression hospitalization model are shown in darker shades of lavender.

Circles denote observed cumulative hospitalizations due to Covid-19 extracted from claims data with the

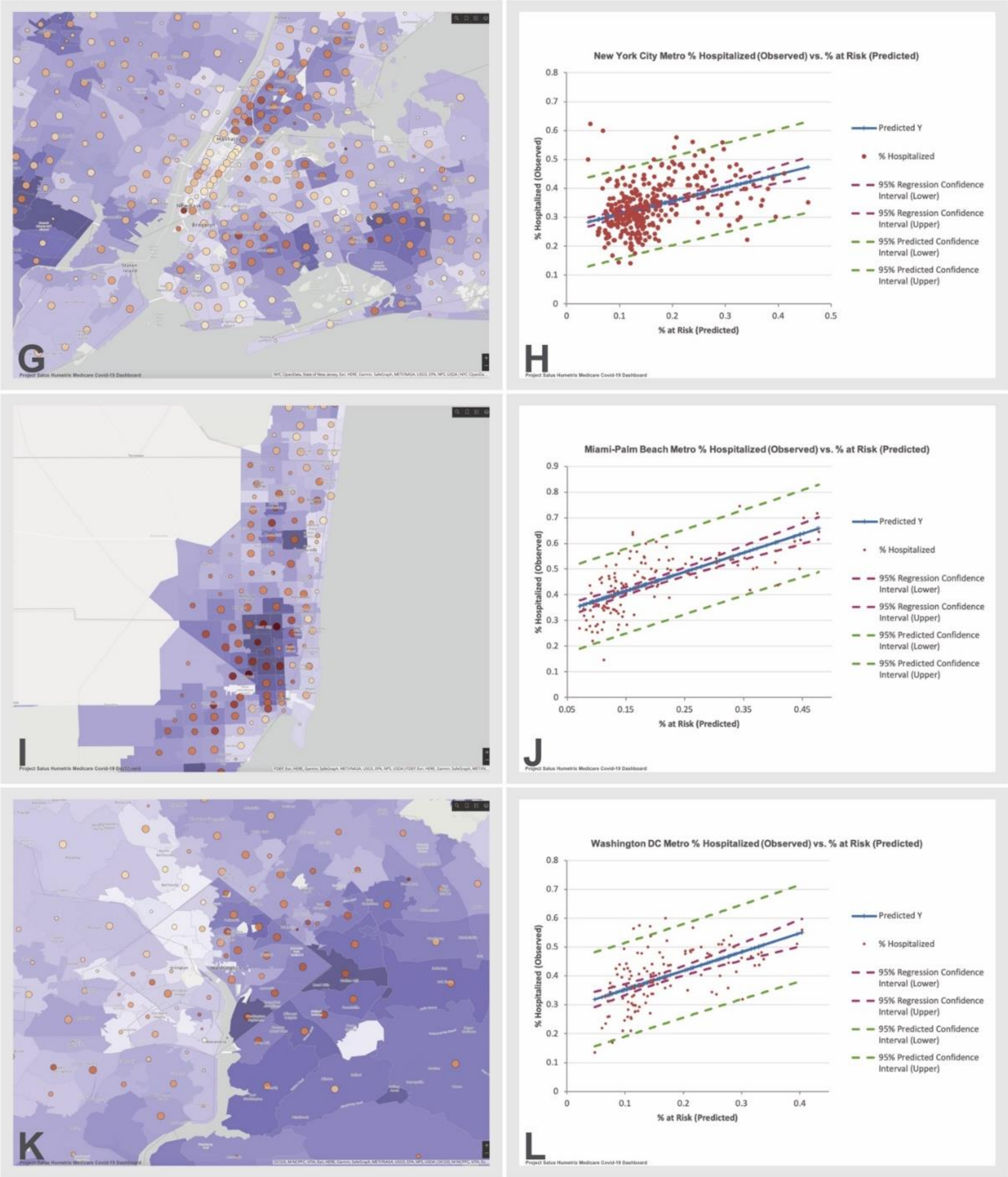

Figure S1. Legend continued: size of the circles scaled to the number of hospitalizations. The percentage of cases requiring hospitalization is displayed by the color of the circles on a beige to dark orange-red color scale with the darker circles indicating higher zip codes with higher percentages of cases requiring hospitalization for Covid-19. Panels B, D, F, H, J, L shows corresponding linear regression analyses of the case hospitalization rates (Y axis) as a function of the risk level in each zip code in each metropolitan region. The following R^2 values for the regressions were: Los Angeles (0.35), Houston (0.44), Phoenix (0.36), New York City (0.16), Miami-Dade-Palm Beach (0.46), Washington DC (0.29). Metro regions zip codes analyzed available on request.

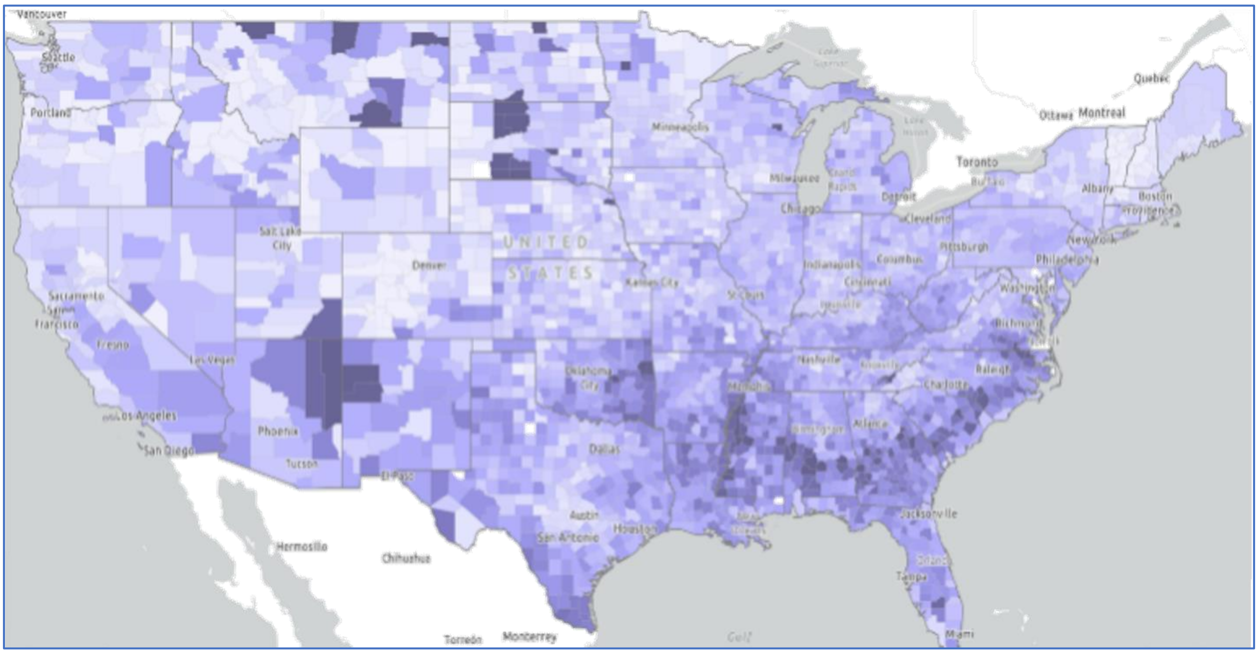

Figure S2. Nationwide county Covid-19 hospitalization risk map shows the percentage of the Salus cohort with a greater than 0.55 predicted probability of hospitalization when diagnosed with Covid-19. The percentage of the cohort in each county at risk is indicated by a light to dark color scale ranging from <8% to >40%. The 25 counties shaded in the darkest color in Montana, North Dakota, South Dakota, Wisconsin, Minnesota, Oklahoma, Nebraska, Utah, Arizona and New Mexico have populations that are on average 68% (Median 69%, IQR 21%) North American Native and 29% (Median 29%, IQR 24%) White.
